# Towards Zero Phrenic Nerve Injury in Reoperative Pediatric Cardiac Surgery: The Value of Intraoperative Phrenic Nerve Stimulation

**DOI:** 10.3390/jcdd11010008

**Published:** 2023-12-28

**Authors:** Sameh M. Said, Ali H. Mashadi, Mahmoud I. Salem, Shanti L. Narasimhan

**Affiliations:** 1 Division of Pediatric and Adult Congenital Cardiac Surgery, Maria Fareri Children’s Hospital, Westchester Medical Center, Valhalla, NY 10595, USA; 2 Department of Cardiothoracic Surgery, Faculty of Medicine, Alexandria University, Alexandria 21544, Egypt; 3 Department of Integrative Biology and Physiology, Undergraduate Studies, University of Minnesota, Minneapolis, MN 55455, USA; masha018@umn.edu; 4 Department of Cardiothoracic Surgery, University of Port Said, Port Said 42526, Egypt; 5 Division of Pediatric Cardiology, University of Minnesota, Minneapolis, MN 55455, USA

**Keywords:** phrenic nerve injury, diaphragm paralysis, diaphragm plication, nerve stimulation, Peña stimulator

## Abstract

Background: Phrenic nerve injury is a devastating complication that results in significant morbidity and mortality. We developed a novel technique to localize the phrenic nerve and evaluate its success. Methods: Two groups of children underwent repeat sternotomy for a variety of indications. Group I (69 patients, nerve stimulator) and Group II (78 patients, no nerve stimulator). Results: There was no significant difference in the mean age and weight between the two groups: (6.4 ± 6.5 years vs. 5.6 ± 6.4 years; *p* = 0.65) and (25.2 ± 24.1 vs. 22.6 ± 22.1; *p* = 0.69), respectively. The two groups were comparable in the following procedures: pulmonary conduit replacement, bidirectional cavopulmonary anastomosis, aortic arch repair, and Fontan, while Group I had more pulmonary arterial branch reconstruction (*p* = 0.009) and Group II had more heart transplant patients (*p* = 0.001). There was no phrenic nerve injury in Group I, while there were 13 patients who suffered phrenic nerve injury in Group II (*p* < 0.001). No early mortality in Group I, while five patients died prior to discharge in Group II. Eleven patients underwent diaphragm plication in Group II (*p* = 0.001). The mean number of hours on the ventilator was significantly higher in Group II (137.3 ± 324.9) compared to Group I (17 ± 66.9), *p* < 0.001. Group II had a significantly longer length of ICU and hospital stays compared to Group I (*p* = 0.007 and *p* = 0.006 respectively). Conclusion: Phrenic nerve injury in children continues to be associated with significant morbidities and increased length of stay. The use of intraoperative phrenic nerve stimulator can be an effective way to localize the phrenic nerve and avoid its injury.

## 1. Introduction

Injury to the phrenic nerve may occur during cardiac surgery and in particular during reoperative settings, where visualization of the nerve may be clouded with dense lesions. The expectation that the phrenic nerve will be present in its exact anatomic location can be misleading, especially during reoperations as the nerve may be displaced by previous conduits, patches, and scar tissue.

The reported incidence of phrenic nerve injury after pediatric cardiac surgery has been 2.2% in some studies [1]. In procedures that require dissection deep in the lung hilum, mobilization of the superior vena cava, and all work on the distal aortic arch, the phrenic nerve is at an even greater risk of injury. 

Phrenic nerve injury is serious, and may result in respiratory insufficiency, the need for repeat intubation, prolonged intensive care unit (ICU) and hospital stay, and potentially the need for additional procedures. From this, it appears that localization of the phrenic nerve with a higher degree of accuracy, particularly in reoperative settings, may enable the potential to prevent its injury. In this manuscript, we present a novel approach to localization of the phrenic nerve in reoperative settings using a hand-held nerve stimulator and aim to evaluate the effectiveness of the approach. 

## 2. Materials and Methods

### 2.1. Patients

A total of 147 patients underwent repeat sternotomy between September 2011 and August 2021. Inclusion criteria were children (<18 years of age) who underwent repeat sternotomy for procedures that either (1) required dissection close to the phrenic nerve, or (2) are known to be associated with phrenic nerve injury. These included distal pulmonary arterial reconstruction, unifocalization, change to the pulmonary conduit, bidirectional cavopulmonary anastomosis, Fontan, aortic arch repair, and heart transplantation.

The patients were divided into two groups, Group I included 69 patients where the technique to localize the phrenic nerve with a nerve stimulator was used, and Group II which included 78 patients where no nerve stimulator was administered.

### 2.2. Nerve Stimulator 

#### 2.2.1. Peña Stimulator

**Figure 1:**
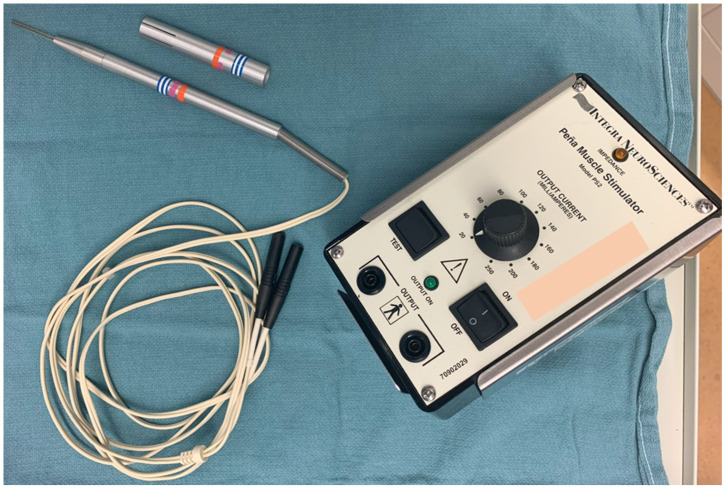
The Peña stimulator provides constant current and was used to localize the phrenic nerve in the current study with a high degree of accuracy.

**Figure 2:**
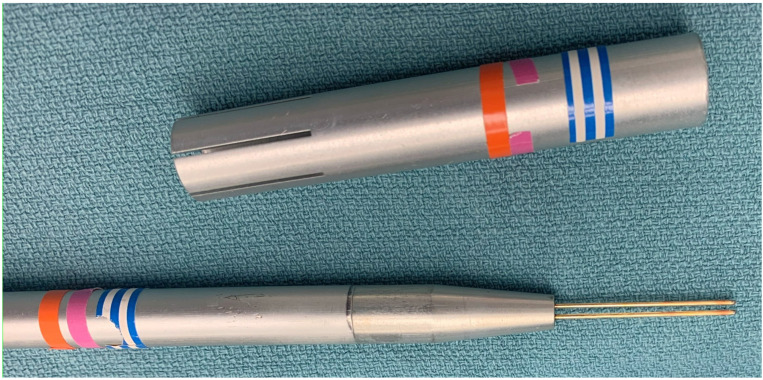
The Peña stimulator has a bipolar hand-held probe which is reusable and easy to use inside the chest cavity.

Alberto Peña pioneered the posterior sagittal anorectoplasty procedure in the 1980s, which required precise localization of the striated muscle structures during the repair [2]. He developed the Peña stimulator (Figure 1, Central Image), which has been used for decades during these procedures. This bipolar hand-held probe (Figure 2) is reusable and easy to use inside the chest cavity to localize the phrenic nerve. We have used the same device for the current study with a high degree of accuracy and success in localizing the nerve.

#### 2.2.2. Disposable Hand-Held Nerve Stimulator 

**Figure 3:**
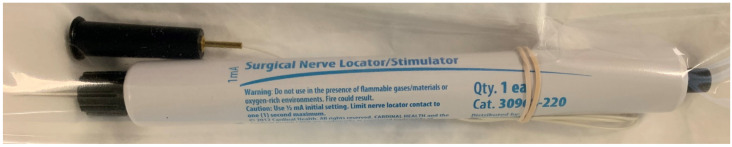
An alternative to the Peña stimulator is a disposable hand-held nerve stimulator that can be used in the same fashion and produces the same results.

Currently, the Peña stimulator is not available in many centers, and is no longer produced, so we have been using a disposable hand-held stimulator recently (Figure 3) that has been as effective as the Peña stimulator in identifying the location of the phrenic nerve intraoperatively. Many other alternatives are also available that serve the same purpose. 

### 2.3. Technique

**Figure 4:**
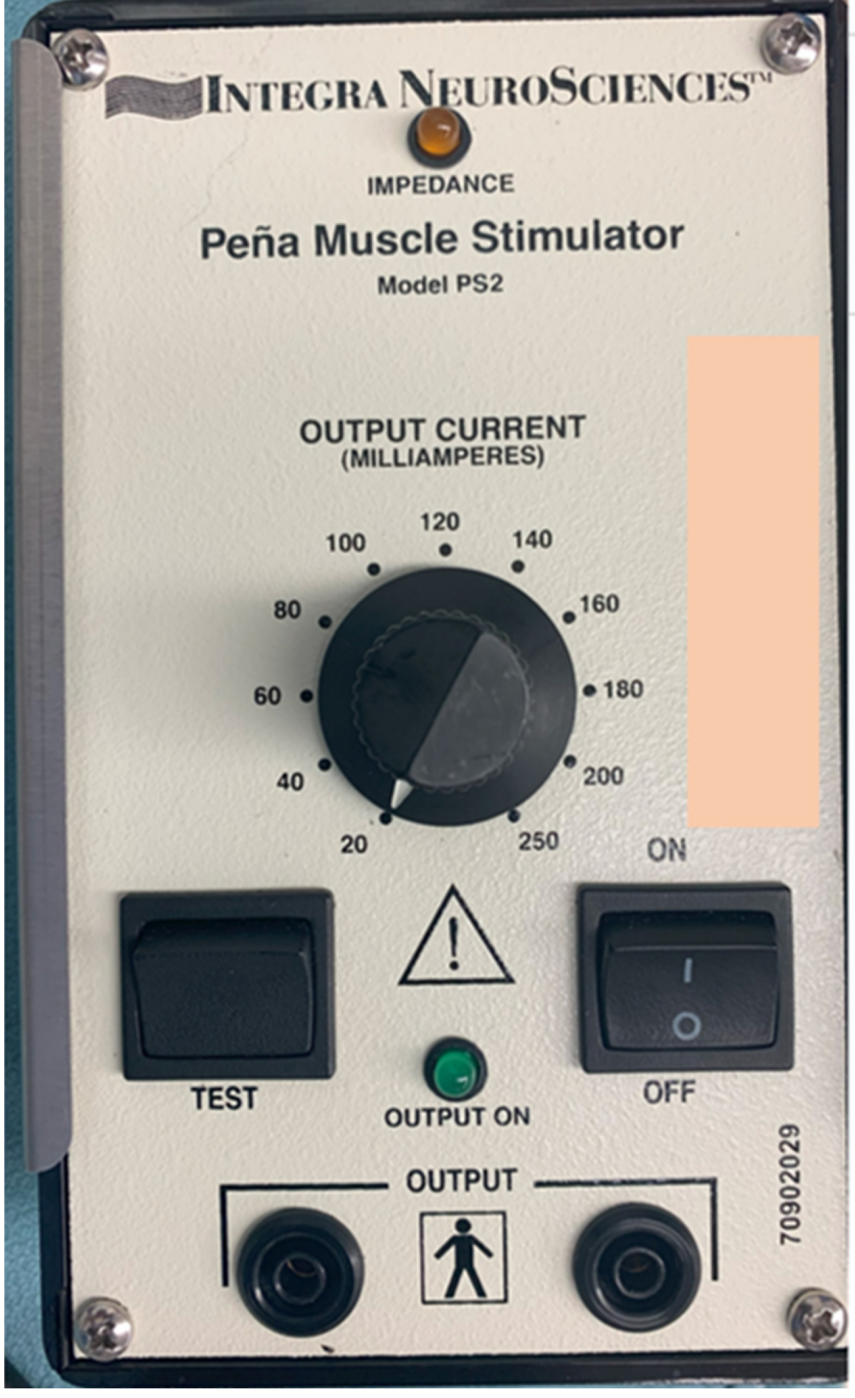
The stimulator/power supply component of the Peña stimulator. The output of the device can be adjusted to achieve the best stimulation at the lowest output.

When using the Peña stimulator, the bipolar probe is passed into the operative field and is connected to the stimulator/power supply (Figure 4), which is usually operated by the anesthesiologist, and its output is adjusted as needed. We do not administer any muscle relaxants during the time when the phrenic nerve is being localized. The power of the stimulator is adjusted, and we trace the location of the phrenic nerve through the constant current that is produced by the stimulator and transferred to the tips of the probe. When the nerve location is accurate, stimulation will result in ipsilateral contraction of the hemidiaphragm, which confirms the location of the nerve, and we mark the entire course through this repeated process (Supplementary Materials Video S1).

At the end of the procedure, we confirm the phrenic nerve integrity once again prior to chest closure. 

### 2.4. Statistical Analysis 

Data were fed to the computer and analyzed using IBM SPSS software package version 20.0. (Armonk, NY, USA: IBM Corp.). Categorical data were represented as numbers and percentages. The Chi-square test was applied to compare between two groups. Alternatively, the Fisher exact correction test was applied when more than 20% of the cells had an expected count less than 5. Continuous data were tested for normality using the Kolmogorov–Smirnov test. Quantitative data were expressed as range (minimum and maximum), mean, standard deviation, and median for quantitative variables that were not normally distributed. The Mann–Whitney test was used to compare two groups. The significance of the obtained results was judged at the 5% level.

## 3. Results

Patients’ characteristics, preoperative diagnoses, operative profiles, and outcomes are shown in Table 1. There were no significant differences in the mean age and weight between Groups I and II: (6.4 ± 6.5 years vs. 5.6 ± 6.4 years; *p* = 0.65) and (25.2 ± 24.1 kg vs. 22.6 ± 22.1 kg; *p* = 0.69), respectively. The two groups were comparable in the following procedures: pulmonary conduit replacement, bidirectional cavopulmonary anastomosis, aortic arch repair, and Fontan procedure. Group I had more pulmonary arterial branch reconstructions (*p* = 0.009) compared to Group II, while Group II had more heart transplantation (*p* = 0.001) compared to Group I.

Intraoperative characteristics were measured, with mean bypass and cross clamp times of 144 and 58 min for Group I, respectively, and a 142-min bypass and 73-min clamp time for Group II. There was no significant difference in the mean number of sternotomies between Group I (2.6 ± 0.89) and Group II (2.5 ± 0.79); (*p* = 0.55).

There was no phrenic nerve injury in Group I, while there were 13 patients who suffered phrenic nerve injury in Group II (*p* < 0.001). The right phrenic nerve was injured in eight patients, while the left was injured in five. Suspicion of phrenic nerve injury was based on elevated hemidiaphragm on routine postoperative chest X-ray and was confirmed by fluoroscopy in the majority of patients (11/13; 84.6%), while ultrasound was the diagnostic modality in two (15.4%). 

No early mortality in Group I, while five patients died prior to discharge in Group II. The mean number of hours on the ventilator was significantly higher in Group II (137.3 ± 324.9 h) compared to Group I (17 ± 66.9 h); (*p* < 0.001). There was no significant difference in the rate of reintubation between the two groups (*p* = 0.30). 

**Figure 5:**
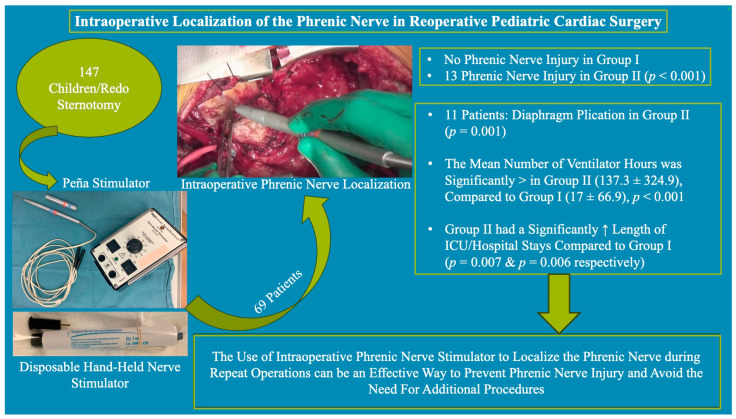
Graphical representation of the method and results in the current study.

Group II had a significantly longer length of stay in the ICU and the hospital compared to Group I, (*p* = 0.007 and *p* = 0.006), respectively (Figure 5). 

Eleven patients required diaphragmatic plication in Group II (*p* = 0.001). Majority of those who required plication were infants (7/11 patients; 63.6%). The mean times for diagnosis of diaphragmatic dysfunction and proceeding with surgical plication of the affected hemidiaphragm were 12 ± 6.75 days and 16 ± 17.81 days, respectively. Late mortality occurred in 5 patients in Group I and 4 in Group II (*p* = 0.73).

## 4. Discussion

The literature has documented varying rates of phrenic nerve injury in congenital cardiac surgery, ranging from 0.3% to 12.8% [3,4]. In our present study, the occurrence of diaphragmatic dysfunction resulting from phrenic nerve injury was observed in 16.7% of cases (13/78 patients), a figure that may seem elevated compared to existing reports. However, when considering our conviction that the actual incidence of phrenic nerve dysfunction is likely underestimated in the literature, coupled with the fact that our study targeted a pediatric demographic undergoing reoperations involving proximity to or direct manipulation of the phrenic nerve, we assert that our sample aligns with the anticipated frequency of injury in such cases.

This incapacitating injury has more serious effects on neonates and infants compared to adults due to the paradoxical motion of the affected hemidiaphragm and the possible contralateral shift of the mediastinum that may occur [5]. Older children and adults may be able to utilize their intercostal muscles for respiration [6]. Infants, on the other hand, have more horizontally oriented ribs with much weaker intercostal muscles. In line with this, in the current study, the majority of those that required surgical plication for paralyzed hemidiaphragm were infants (7/11 patients; 63.6%). 

Phrenic nerve injury affects both the ipsilateral diaphragm due to its paradoxical movement, resulting in a decrease in its functional residual capacity with subsequent atelectasis, retained secretions, pneumonia, and repeat intubation, and the contralateral side, which can be affected by the mediastinal shift that may occur [7].

Certain cardiac procedures are known to be associated with higher risk of phrenic nerve injury. While it is less common for such injury to occur with primary sternotomy, the phrenic nerve remains vulnerable during procedures that require harvesting of autologous pericardium and thymectomy such as arterial switch and the repair of total anomalous pulmonary venous connections (TAPVC). In the study by Greene and colleagues, the authors reported an incidence of 4.8% of phrenic nerve injury after surgery for pulmonary atresia, ventricular septal defect, and major aortopulmonary collaterals (MAPCAs) [8]. All patients who suffered diaphragmatic paralysis underwent surgical plication of the affected hemidiaphragm after a median time interval of two days; however, the median interval for recognition of the injury was 11 days. The authors proposed a few mechanisms that may explain phrenic nerve injury after this procedure such as: the thymic gland resection that is required during the intervention, autologous pericardium harvest, dissection of the MAPCAs, stretch injury that may occur during the reconstruction, and potentially central line placement. Despite plicating all patients who suffered the injury in a relatively quick and aggressive fashion, clinical improvement occurred in only 75% of affected individuals. 

In the setting of reoperation, this becomes a bit more challenging because the ability of the surgeon to visualize the nerve with accuracy is reduced and the location of the nerve may change based on the burden of scar tissue, the degree of adherence to previous pulmonary conduits, and/or pulmonary/aortic arch patches. 

Unfortunately, the difficulty in recognizing this injury after pediatric cardiac surgery may result in a delay in intervention, leading to other morbidities related to prolonged/repeat intubation. The typical manifestations include the inability to wean off mechanical ventilation, failure of extubation, or the need for repeat intubation. Associated morbidities include recurrent pneumonia and atelectasis, which may further complicate the clinical settings [9].

Early diagnosis of phrenic nerve injury is key to shortening the length of stay and avoiding many of the morbidities that are associated with delayed recognition of this complication. Elevated hemidiaphragm on routing chest X-ray should raise clinical suspicion; however, this will not be easily recognized for intubated patients. Confirmation of the diagnosis of phrenic nerve injury requires ultrasound, fluoroscopy, and/or electromyography [10]. In the current study, diagnosis and confirmation of phrenic nerve injury followed the same algorithm. 

Standard management of diaphragmatic dysfunction includes prolonged ventilatory support and/or diaphragmatic plication. Diaphragm recovery has been reported in the literature after phrenic nerve injury and after diaphragmatic plication as well. However, the interval to recovery has been variable [11]. In the study by Simansky and colleagues, the mean time to extubation was 40.8 days [12]. While diaphragmatic recovery may occur with conservative measures, early diaphragm plication facilitates earlier extubation and helps avoid complications related to prolonged ventilatory support. It remains unclear what the optimal timing for diaphragmatic plication is. Those who advocate for late plication argue that spontaneous recovery of the diaphragmatic function may occur after a period of two weeks; on the other hand, early plication proponents believe that plication should be done as soon as the diaphragmatic paralysis is confirmed [13,14]. In some studies, delayed plication beyond 10 days has been associated with increased risks of postoperative pneumonia and death [15]. Aside from those that support late and early plication, a third group prefers to make the decision for diaphragmatic plication based upon the clinical and respiratory status of the patient. The mean time from surgery to the diagnosis of diaphragmatic paralysis in the current study was 12 ± 6.75 days, while the mean time from diagnosis of diaphragmatic paralysis to surgical plication of the hemidiaphragm was 16 ± 17.81 days. 

Although spontaneous recovery has been reported, it is quite rare. Waiting after confirming diaphragmatic dysfunction results in a prolonged need for ventilatory/respiratory support, recurrent intubation, increased risk of postoperative pneumonia, prolonged ICU and hospital stay, and even mortality. In a multicenter study of 112,110 patients from 43 hospitals through the pediatric health information system database, the authors found a 2.2% overall incidence of diaphragmatic paralysis, with 24% (603 patients) requiring plication [1]. The authors also noted that infants under one month of age exhibited the greatest requirement for plication, and conversely, as the child’s age increased, the likelihood of necessitating plication diminished. In another large study by Floh and colleagues, the authors found that younger age and the use of deep hypothermic circulatory arrest were the two major predictors for diaphragmatic dysfunction [16]. 

Due to the known morbidities and possible mortalities associated with phrenic nerve injury, especially in children, we developed a novel strategy to localize the phrenic nerve intraoperatively with a high degree of accuracy, in particular during reoperative settings with the goal of minimizing, if not preventing, such devastating injury. The Peña stimulator has been used for decades to precisely localize the striated muscles during repair of anorectal malformation. We have utilized this device, which provides a constant current to define the course of the phrenic nerve during repeat operations in children, particularly in procedures that require working in close vicinity of the phrenic nerve such as distal pulmonary arterial branches reconstruction, unifocalization procedures, pulmonary conduit replacement, and those that require extensive mobilization of the superior and inferior venae cavae such as bidirectional cavopulmonary anastomosis and Fontan procedure. We were able to precisely locate the phrenic nerve, resulting in no injury or any diaphragmatic dysfunction postoperatively. This has been our standard practice in the last few years. 

Other forms of nerve stimulator can be used, as we indicated in our method section, because the Peña stimulator is no longer produced. We believe that this technique is easy to use, is reproducible, and has a high degree of accuracy in identifying the phrenic nerve intraoperatively, with specific value in reoperative settings where surgical visualization is obscured by scar tissue and previous adhesions. 

## 5. Conclusions

In conclusion, despite all the advances in surgical techniques and postoperative care, phrenic nerve injury continues to occur, and while the reported incidence in the literature may be underestimated, it continues to be one of the most devastating complications after cardiac surgery, especially in children.

Preventing this injury is paramount and utilizing a technique such as intraoperative phrenic nerve localization, provides an accurate and effective way to achieve this goal. 

## Figures and Tables

**Table 1:** Patients’ Characteristics.

	Group I (*n* = 69) “Nerve Stimulation”	Group II (*n* = 78) “No Nerve Stimulation”	Test of Significance	*p*
**Age at time of surgery (years)**				
*Mean ± SD.*	6.4 ± 6.5	5.6 ± 6.4	U = 2574.50	0.651
*Median (Min.–Max.)*	4 (0.05–19.2)	3 (0.06–31)		
**Weight at time of surgery (kg)**				
*Mean ± SD.*	25.2 ± 24.1	22.6 ± 22.1	U = 2254.50	0.694
*Median (Min.–Max.)*	13.8 (1.7–103.4)	13.2 (4–87)		
**Genetic syndromes**	8 (11.6%)	9 (11.5%)	χ^2^ = 0.0	0.992
**Diagnosis**				
*Shone’s complex*	2 (2.9%)	3 (3.8%)	χ^2^ = 0.100	^FE^*p* = 1.000
*Heterotaxy*	4 (5.8%)	2 (2.6%)	χ^2^ = 0.977	^FE^*p* = 0.420
*TAPVC*	1 (1.4%)	4 (5.1%)	χ^2^ = 1.508	^FE^*p* = 0.371
*Interrupted aortic arch*	1 (1.4%)	6 (7.7%)	χ^2^ = 3.146	^FE^*p* = 0.121
*Pulmonary Atresia*	12 (17.4%)	6 (7.7%)	χ^2^ = 3.205	0.073
*Hypoplastic Left Heart*	7 (10.1%)	10 (12.8%)	χ^2^ = 0.256	0.613
**Procedure**				
*RV-PA conduit Replacement*	10 (14.5%)	12 (15.4%)	χ^2^ = 0.023	0.880
*Bidirectional Glenn*	3 (4.3%)	7 (9%)	χ^2^ = 1.236	^FE^*p* = 0.336
*Pulmonary valve Replacement*	10 (14.5%)	1 (1.3%)	χ^2^ = 9.230 *	0.002 *
*Aortic Arch Repair*	3 (4.3%)	1 (1.3%)	χ^2^ = 1.300	^FE^*p* = 0.342
*Fontan*	4 (5.8%)	7 (9%)	χ^2^ = 0.534	0.465
*Heart transplant*	2 (2.9%)	17 (21.8%)	χ^2^ = 11.616 *	0.001 *
**Number of sternotomies**				
*Mean ± SD.*	2.6 ± 0.89	2.5 ± 0.79	U = 2558.50	0.550
*Median (Min.–Max.)*	2 (2–5)	2 (2–5)		
**Intraoperative characteristics (min)**			-	-
*CPB Mean (Min.–Max.)*	144 (0–453)	142 (0–422)		
*AXC Mean (Min.–Max.)*	58 (0–220)	73 (0–254)		
**Concomitant procedures**				
*PA branch reconstruction*	33 (47.8%)	21 (26.9%)	χ^2^ = 6.883 *	0.009 *
*Unifocalization*	1 (1.4%)	0 (0%)	χ^2^ = 1.138	^FE^*p* = 0.469
**SVC cannulation**	3 (4.3%)	9 (11.5%)	χ^2^ = 2.525	0.112
**Hours on ventilator**				
*Mean ± SD.*	17 ± 66.9	137.3 ± 324.9	U = 1809.50 *	<0.001 *
*Median (Min.–Max.)*	0 (0–480)	0 (0–1680)		
**Re-intubation**	7 (10.1%)	12/75 (16%)	χ^2^ = 1.076	0.300
**Right phrenic nerve injury**	0 (0%)	8 (10.3%)	χ^2^ = 7.484 *	^FE^*p* = 0.007 *
**Left phrenic nerve injury**	0 (0%)	5 (6.4%)	χ^2^ = 8.481 *	^FE^*p* = 0.003 *
**Total phrenic nerve injury**	0 (0%)	13 (16.7%)	χ^2^ = 12.616 *	<0.001 *
**LOS ICU**				
*Mean ± SD.*	17 ± 31.1	61.6 ± 98.7	U = 1976.50 *	0.007 *
*Median (Min.–Max.)*	5 (1–173)	11 (1–489)		
**LOS hospital**				
*Mean ± SD.*	21.3 ± 33.1	69.1 ± 103.4	U = 1977.50 *	0.006 *
*Median (Min.–Max.)*	9 (2–173)	18.5 (3–565)		
**Plication of diaphragm**	0 (0%)	11 (14.1%)	χ^2^ = 10.518 *	0.001 *
**Early mortality**	0 (0%)	5 (6.4%)	χ^2^ = 4.579	^FE^*p* = 0.061
**Late mortality**	5 (7.2%)	4 (5.1%)	χ^2^ = 0.286	^FE^*p* = 0.735

TAPVC: total anomalous pulmonary venous connection; RV: right ventricular; PA: pulmonary artery; SVC: superior vena cava; LOS: length of stay; SD: Standard deviation; U: Mann–Whitney test; χ^2^: Chi-square test; FE: Fisher exact; *p*: *p* value for comparing between the two studied groups; *: Statistically significant at *p* ≤ 0.05.

## Data Availability

The data presented in this study are available on request from the corresponding author upon reasonable request.

## Supplementary Materials

The following supporting information can be downloaded at: https://www.mdpi.com/article/10.3390/jcdd11010008/s1; Video S1: Operative demonstration phrenic nerve stimulation using the Peña stimulator during a reoperative setting.
